# Autovaccine immunoprophylaxis in patients with neurogenic bladder experiencing recurrent urinary tract infections

**DOI:** 10.3389/fimmu.2025.1626422

**Published:** 2025-08-01

**Authors:** Miguel Ángel Bonillo-García, Domingo Guzmán Ordaz-Jurado, Juan Ortiz-Salvador, Josep Oriol Colet-Guitert, Eduardo Morán-Pascual, Esther Martínez-Cuenca, Salvador Arlandis-Guzmán

**Affiliations:** Sección de Urología Reconstructiva y Funcional, Servicio de Urología, Hospital Universitario y Politécnico La Fe, Valencia, Spain

**Keywords:** autovaccination, emergency admissions, hospitalization, neurogenic bladder, urinary tract infections

## Abstract

**Introduction:**

Neurogenic Bladder (NB) patients are highly susceptible to recurrent urinary tract infections (UTIs), often requiring repeated hospitalizations and prolonged antibiotic use. Current preventive strategies, including long-term antibiotics, frequently fail due to resistance and limited efficacy. Autovaccination, a form of personalized immunoprophylaxis using inactivated patient-specific bacterial strains, has shown potential in reducing UTI recurrence but has not been well studied in NB populations.

**Methods:**

A prospective, single-center study was conducted at the Hospital Universitari i Politécnic La Fe València, Spain. Eligible participants were individuals of any gender, aged between 18 and 65 years, with a confirmed diagnosis of NB. They were required to have experienced recurrent UTIs within the past twelve months, despite having undergone a six-month regimen of prophylactic antibiotics without success. Participants received a sublingual bacterial autovaccine (Uromune^®^) prepared from uropathogens isolated from their own urine cultures. Primary outcomes included changes in hospitalization and emergency admission rates, while secondary outcomes assessed UTI-free time and patient-reported outcomes using validated instruments.

**Results:**

The study included 71 adult NB patients with recurrent UTIs. Autovaccination significantly reduced hospitalizations (from 1.76 ± 3.47 to 0.78 ± 1.38, p < 0.001) and emergency admissions (from 8.62 ± 6.35 to 3.93 ± 4.48, p < 0.001). At 3, 6, 9, and 12 months post-treatment, UTI-free rates were 69.1%, 42.6%, 29.4%, and 20.6%, respectively. Most patients reported high satisfaction and perceived clinical improvement.

**Conclusions:**

Autovaccination appears to be a promising strategy for reducing the burden of recurrent UTIs in NB patients, with high patient satisfaction and fewer hospital visits. These findings support the need for larger, multicenter trials to confirm efficacy and define optimal treatment protocols.

## 1 Introduction

Neurogenic Bladder (NB), also known as Neurogenic Lower Urinary Tract Dysfunction, (NLUTD) manifests when an individual loses bladder control due to any disturbance in the brain, spinal cord, or nerves, which leads to improper storage and release of urine (1).

Patients with NB face a heightened risk of recurrent urinary tract infections (UTIs) owing to incomplete bladder emptying, vesicoureteral reflux, prolonged catheter use (2). In particular cases of spinal cord injury (SCI) patients, SCI-induced immunodepression, premature onset of immune frailty, and the impaired local immune response contribute to the increased susceptibility to UTIs (3). Hence, this frequent medical assistance, increased antibiotic use, and a higher risk of antibiotic resistance (4). This leads to rising healthcare costs and limited treatment options. Additionally, persistent infections contribute to chronic inflammation, renal function decline, and overall deterioration in patient well-being (4).

Current treatment strategies for recurrent UTIs in NB patients include antibiotic therapy, bladder management, and prophylactic measures such as low-dose antibiotics, urinary acidification, and probiotics (5). Finally, these approaches often fail due to antibiotic resistance and limited long-term efficacy (5).

Given the increasing challenges of managing recurrent UTIs in this population, there is an urgent need for alternative preventive strategies. Bacterial sublingual vaccine, a personalized immunoprophylactic approach using inactivated pathogens isolated from the own infection of the patient, has shown promise in the treatment of recurrent UTIs in general populations. However, it has not been extensively studied in NB patients (6–10). MV140, commercially known as Uromune^®^, is a multivalent bacterial vaccine used to prevent and treat recurrent UTIs. It was approved by the Spanish Agency for Medicines and Health Products (AEMPS) in October 2010. Since January 2018, its use has become more relevant in Spain after new regulations limited the use of polyvalent vaccines made from collection strains. The AEMPS and the General Council of Pharmacists now allow only autovaccines—like MV140—prepared from bacteria isolated from each patient. This restriction is due to a regulatory change requiring specific authorization for vaccines with industrialization processes different from those of autovaccines.

Over the past ten years, numerous studies have demonstrated the effectiveness and efficiency of MV140 with its high tolerability and minimal side effects (11–33). Research on MV140 has also documented significant clinical improvements across diverse patient demographics, encompassing various ages, sexes, and pre-existing conditions. This includes individuals with autoimmune diseases undergoing immunosuppressive therapy, patients experiencing recurrent genital candidiasis, smokers, and those affected by neurogenic bladders, chronic prostatitis, chronic renal disease, renal transplants, and lymphoproliferative disorders. Additional findings include positive outcomes for women post-trans-obturator tape surgery, children with urological renal complications, and elderly patients (11–33). In March 2023, the EAU Guidelines on Urological Infections acknowledged the advantages and safety of MV140 as a polybacterial sublingual vaccine and indicated that it has proven more effective than placebo in reducing the frequency of female recurrent infections, recommending the use of immunoactive prophylaxis to reduce recurrent UTI in all age groups (34).

Although immunoprophylaxis had been tested previously in NB patients with recurrent UTIs (35–37), the only publication regarding the use of MV140 in this population is a pilot study that we conducted and presented in 2014 as a congress communication. It reported the use of MV140 in a cohort of 17 patients with NB, observing a decrease in the UTI rate from a median of 4 per year before vaccination to 0 per year after vaccination (p = 0.9) (38).

Hence, this study aims to contrast the previous results and expand the knowledge of treatment options for the NB population suffering from recurrent UTIs by exploring the potential of Uromune^®^ autovaccine-based immunoprophylaxis in a larger population. The primary objective of this research is to determine whether autovaccine immunoprophylaxis can reduce hospital admissions and emergency room visits in patients with NB, and the secondary goal is to evaluate patient satisfaction with this therapy.

## 2 Materials and methods

### 2.1 Design and patients

This study was designed as a monocenter, prospective, trial conducted at the Hospital Universitari i Politécnic La Fe València in Valencia, Spain.

Eligible participants were enrolled from May 2017 to July 2021, individuals of any gender, aged between 18 and 65 years, with a confirmed diagnosis of NB based on urodynamic studies. They were required to have experienced recurrent UTIs over the past twelve months despite failing a six-month regimen of prophylactic antibiotics. UTI was diagnosed based on the presence of significant bacteriuria, defined as >10² CFU/mL when performing clean intermittent catheterization (CIC), >10^4^ CFU/mL in clean-voided specimens, and any detectable concentration in suprapubic aspirates (39).Participants had to be able to attend medical consultations, adhere to the treatment plan, and be free from UTIs at the time of inclusion. A minimum follow-up period of twelve months was necessary for inclusion. Exclusion criteria included immunocompromised or immunosuppressed individuals, including those with severe diabetes, hematological diseases, or undergoing chemotherapy or immunosuppressive treatments. Other exclusion factors included urethral strictures, chronic bacterial prostatitis, previous bladder reconstruction surgery such as augmentation cystoplasty, and infections caused by multidrug-resistant organisms. Patients with poor compliance, those receiving another form of immunotherapy, individuals with urinary tract stones, and those who were pregnant, postpartum, or breastfeeding at the time of enrollment were also excluded. Patients requiring antibiotics for any other reason unrelated to UTIs tract were not eligible for the study.

The study adhered to the ethical principles outlined in the Declaration of Helsinki and received approval from the Hospital Universitari i Politécnic La Fe ethical committee (2024-0743-1) prior to its initiation. It also complied with the EU General Data Protection Regulation (GDPR); all personal identifiers were removed from the findings. Informed consent was obtained from all participants or their legal representatives.

### 2.2 Treatment

Bacterial sublingual autovaccine (Uromune^®^, Q Pharma/Inmunotek laboratories in Alicante, Spain) is composed of whole-cell, heat-inactivated bacteria (300 Formazin Turbidity Units) isolated from the urine sample of each patient, suspended in a mixture of glycerol, sodium chloride, artificial pineapple flavoring, and water.

Urine samples were collected from midstream first-morning voiding (spontaneous voiding) or catheterized bladder (CIC patients), with microorganisms identified via standard techniques (phenotypic/biochemical tests). The autovaccine contained 100% single strain or 50%/50% for two strains.

The treatment regimen consisted of two 100μL sublingual puffs daily (10^8^ heat-inactivated bacteria/puff) for 3 months. Patients avoided eating/drinking for 30 minutes post-dose, and treatment was paused during febrile episodes.

### 2.3 Objectives and variables

In terms of demographics and clinical characteristics, the study investigated sex, age, the etiology of the neurogenic bladder, and catheterization methods. At baseline, the infection characteristics of the patients included the previous number of UTIs and the composition of the bacterial cultures from urine samples.

The primary objective was assessed through the number of hospitalizations and emergency admissions before and after autovaccination. Secondary objectives included monitoring the number of UTIs post-vaccination and conducting validated patient-reported outcomes (PROs) to evaluate patient satisfaction, including the Benefits, Satisfaction and Willingness (BSW) questionnaire (40), the Treatment Benefit Scale (TBS) (41), and a Satisfaction Visual Analogic Scale (VAS; 0 to 100). All PROs were administered by the authors at the end of the follow-up period and completed by the patients on the same day as the final visit.

UTI-free time was defined as the duration in which the patient experienced no new or worsening urinary symptoms consistent with infection. While urinalysis and urine cultures were obtained when clinically indicated, asymptomatic bacteriuria was not considered a UTI.

### 2.4 Statistics

Categorical data were presented as absolute numbers and percentages, while continuous variables were reported as averages with standard deviations. For parameters with missing data, calculations were performed using only the available values.

Statistical analysis included various tests such as the Mann-Whitney test, Fisher’s exact test, and Pearson’s chi-squared test.

Statistical significance was defined as a p-value of less than 0.05, and all statistical tests were two-sided.

All statistics were performed using GraphPad statistical software version 5.0 (GraphPad Software, Inc., La Jolla, CA, USA).

## 3 Results

### 3.1 Baseline demographic and clinical characteristics

A total of 70 patients with NB and recurrent UTIs were evaluated (**Table 1**). The mean age of the participants was 52.8 years, with a standard deviation (SD) of 14.1 years. The majority of the patients were men, representing 68.6% (n=48) of the cohort.

**Table 1:** Demographic and clinical characteristics at baseline.

Age (years), mean (SD)	52.8 (14.1)
Men, n (%)	48 (68.6)
Etiology, n (%)	
Spinal cord injury	34 (48.68)
Myelomeningocele	14 (20.0)
Multiple sclerosis	6 (9.2)
Other	17 (7.4)
Bladder management, n (%)	
Void	7 (10)
Clean Intermittent Catheterization	48 (68.6)
Indwelling Urinary Catheter	15 (21.4)

SD, Standard Deviation.

The underlying etiology of NB varied among participants. Spinal cord injury was the most common cause, observed in 33.8% of patients (n=22). Other frequent causes included myelomeningocele (20.0%, n=13) and traumatic spinal cord injury (13.8%, n=9). Less common etiologies included multiple sclerosis (9.2%, n=6), and vascular spinal cord injury (3.1%, n=2). Additionally, several conditions contributed to NB in a small proportion of patients, with each accounting for 1.5% of cases.

Regarding bladder management, the majority of patients used clean intermittent catheterization (68.6%, n=48), while 7 (10%) void and 15 (21.4%) used an indwelling urinary catheter.

### 3.2 Infection characteristics at baseline

The infection characteristics of the study population at baseline are listed in the **Table 2**. The mean number of UTIs experienced by patients in the 12 months before the study was 11.2, with an SD of 6.1. The distribution of UTI frequency showed that most patients (73.9%, n=51) had experienced >6 UTIs in the preceding year. A smaller proportion of patients reported 4–6 UTIs (21.7%, n=15), while only 4.3% (n=3) had experienced 1–3 UTIs.

**Table 2:** Infection characteristics of the patients at baseline.

Number of UTI pre, mean (SD)	11.2 (6.1)
1-3, n (%)	3 (4.3)
4-6, n (%)	15 (21.7)
>6, n (%)	51 (73.9)
Number of microorganisms, n (%)	1.4 (0.6)
1	47 (67.1)
2	21 (30.0)
3	0 (0)
4	2 (2.9)
Species isolated for the autovaccine	
*Escherichia coli*	36
*Klebsiella pneumoniae*	18
*Pseudomonas aeruginosa*	10
*Enterococcus faecalis*	9
*Proteus mirabilis*	8
*Enterobacter cloacae*	2
*Citrobacter koseri*	1
*Staphylococcus epidermidis*	1
*Candida* sp.	1
*Pseudomonas* sp.	1
*Klebsiella oxytoca*	1
*Enterobacter aerogenes*	1
*Morganella morganii*	1
*Stenotrophomonas maltophilia*	1

UTI, Urinary Tract Infection.

The mean number of microorganisms isolated in the urine sample collected for the autovaccination was 1.6, with an SD of 0.7. A single microorganism caused most previous infections (67.1%, n=47), while 30.0% (n=21) involved two microorganisms and 2.9% four microorganisms (n=2). Infections involving three microorganisms were not reported.

The most frequently isolated microorganism was *Escherichia coli*, found in 36 cultures. This was followed by *Klebsiella pneumoniae* (18 cultures), *Pseudomonas aeruginosa* (10), *Enterococcus faecalis* (9), and *Proteus mirabilis* (8). Less frequently isolated organisms included *Enterobacter cloacae* (2 cultures), *Citrobacter koseri* (1), *Staphylococcus epidermidis* (1), *Candida* sp. (1), *Pseudomonas* sp. (1), *Klebsiella oxytoca* (1), *Enterobacter aerogenes* (1), *Morganella morganii* (1), and *Stenotrophomonas maltophilia* (1).

### 3.3 Hospitalizations and emergency admissions after and before autovaccination

**Figure 1:**
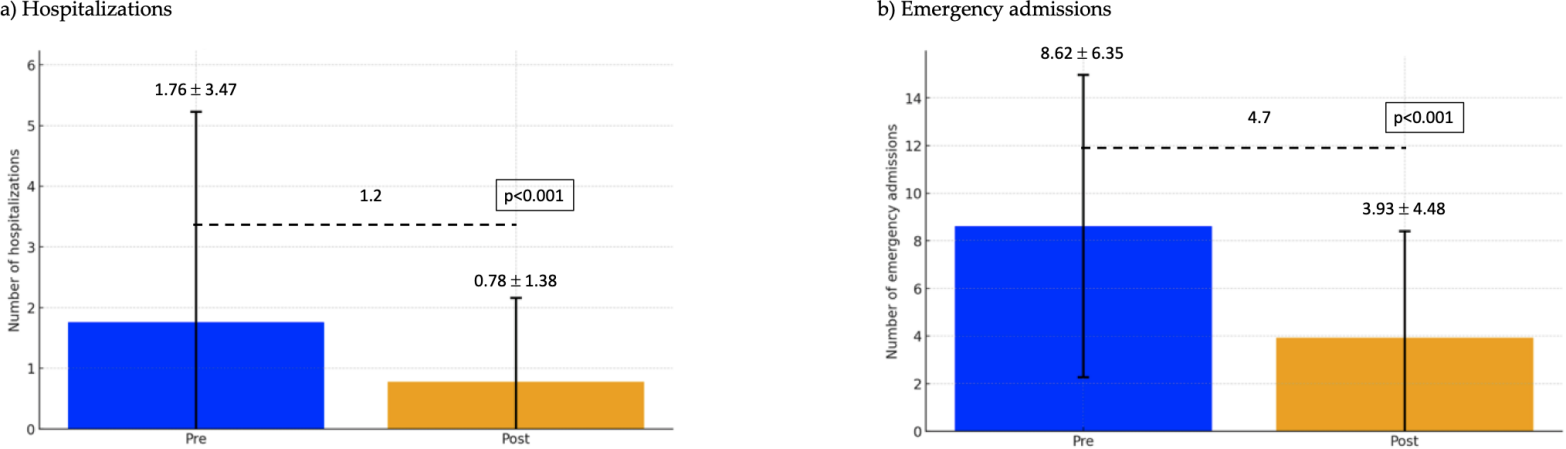
Hospitalizations and emergency admissions pre and post autovaccination. **(a)** Hospitalizations. **(b)** Emergency admissions.

The results shown a significant reduction in the number of hospitalizations following autovaccination (**Figure 1a**). The mean number of hospitalizations decreased from 1.76 ± 3.47 to 0.78 ± 1.38, with a mean difference of 1.2 hospitalizations and a p-value < 0.001, indicating statistical significance.

Similarly, there was a marked decrease in emergency admissions (**Figure 1b**). The mean number of emergency admissions dropped from 8.62 ± 6.35 to 3.93 ± 4.48, with a mean reduction of 4.7 visits. The statistical significance of this difference is confirmed by a p-value < 0.001.

### 3.4 Time of UTI-free status after autovaccination

**Figure 2:**
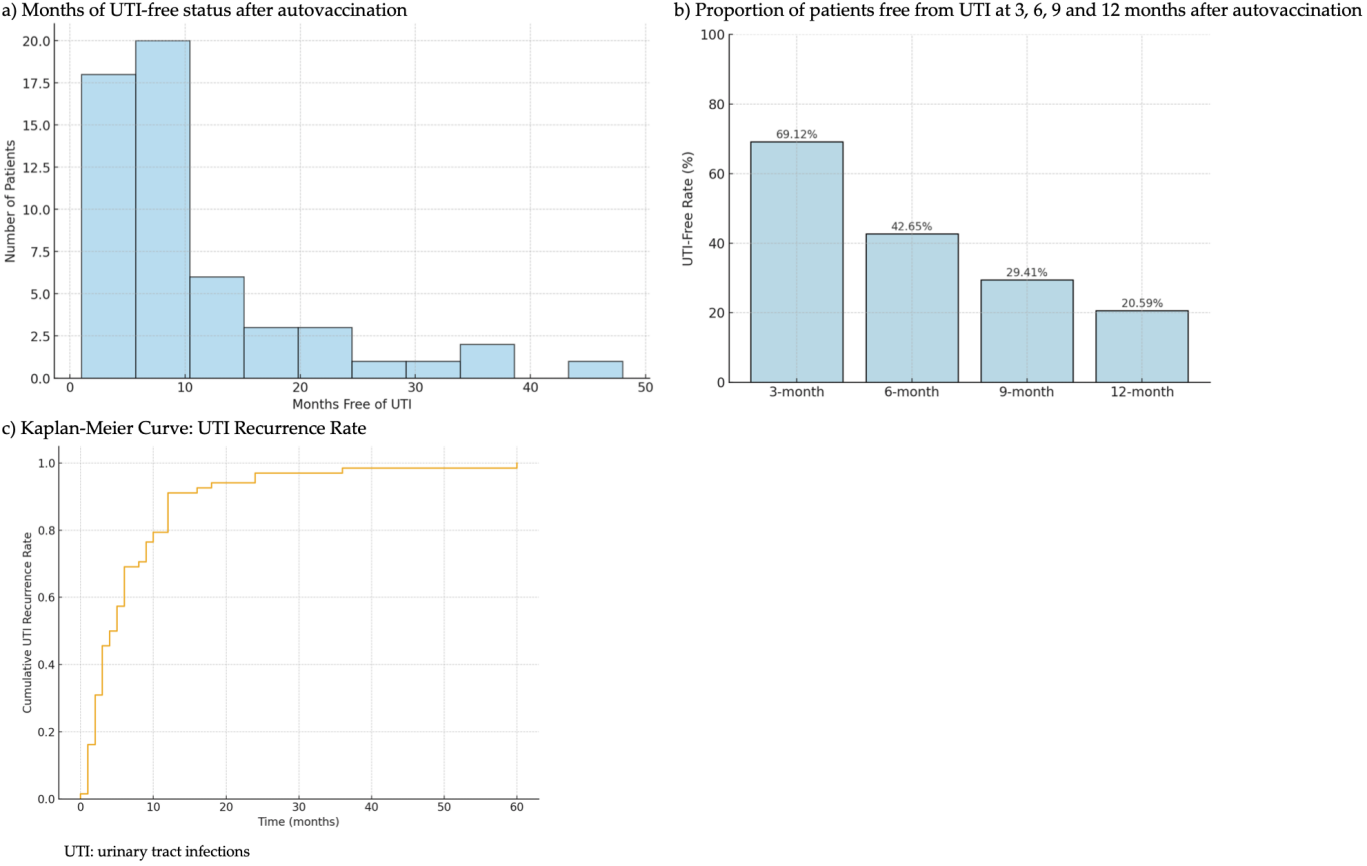
Time of UTI-free status after autovaccination. **(a)** Months of UTI-free status after autovaccination. **(b)** Proportion of patients free from UTI at 3, 6, 9 and 12 months after autovaccination. **(c)** Kaplan-Meier Curve: UTI Recurrence Rate.

**Figure 2** illustrates the time of UTI-free status after autovaccination, including a Kaplan-Meier Curve of the UTI recurrence rate (**Figure 2c**). The mean of UTI-free time was 10.62 months, with an SD of 10.04 months (**Figure 2a**). At 3 months after autovaccination, 69.12% of patients remained free from UTI. This rate declined to 42.65% at 6 months, 29.41% at 9 months, and 20.59% at 12 months (**Figure 2b**).

### 3.5 Patient-reported outcomes after autovaccination

**Figure 3:**
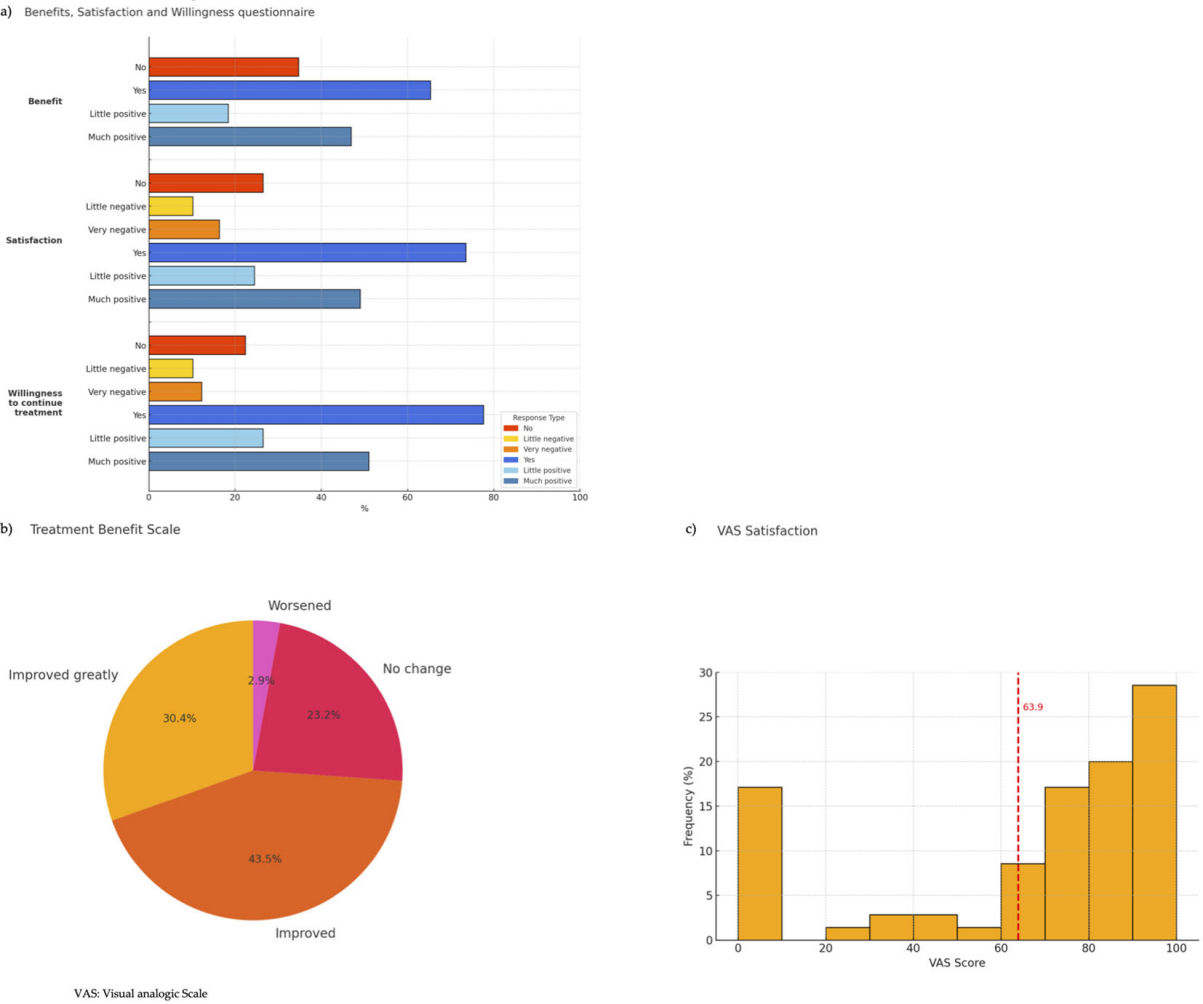
Patient-reported outcomes. **(a)** Benefits, Satisfaction and Willingness questionnaire **(b)** Treatment Benefit Scale **(c)** VAS Satisfaction.

Regarding PROs, responses to the BSW questionnaire showed that the majority of patients (24, 49.0%) reported being “much satisfied,” as shown in **Figure 3a**. Additionally, 12 patients (24.5%) indicated they were “less satisfied.” A smaller segment of patients expressed dissatisfaction, with 5 subjects (10.2%) “less dissatisfied” and 8 (16.3%) “much dissatisfied.” Regarding the willingness to 204 undergo treatment from the BSW questionnaire, more than half of the patients (25, 51.0%) reported being “much willing”, and 13 (26.5%) were “less willing” (**Figure 3a**). Conversely, a smaller segment showed hesitancy, with 5 (10.2%) being “less unwilling” and 6 (12.2%) “much unwilling”. In terms of perceived benefit, 23 patients (46.9%) reported experiencing “much benefit,” while 9 (18.4%) noted “little benefit.” However, 17 patients (34.7%) reported no perceived benefit from the treatment.

The TBS results showed that most participants reported a positive effect from the treatment (**Figure 3b**). Specifically, the majority indicated that their condition had either “Improved greatly” (21, 30.4%) or “Improved” (30, 43.5%). A smaller portion reported “No change” (16, 23.2%) and only a very limited number indicated that their condition had “Worsened” (2, 2.9%).

The VAS satisfaction score distribution shows a concentration of scores around 70–100, with an average of 63.9, indicating moderate to high overall satisfaction among participants (**Figure 3c**).

### 3.6 Association of parameters with hospitalizations and emergency admissions change after autovaccination

The analysis of associations between clinical parameters and changes in hospitalizations and emergency admissions after autovaccination is shown in **Table 3**. Most variables displayed no significant correlation with a reduction in hospitalizations. A significant correlation was observed solely between the number of UTIs in the previous year and hospitalizations, with a Pearson correlation of 0.332 and a p-value of 0.005.

**Table 3:** Association of parameters with hospitalizations and emergency admissions change after autovaccination.

Variable 1	Variable 2	Pearson correlation	Pearson p-value	Spearman correlation	Spearman p-value	T-test statistic	T-test p-value	Conclusion
Number of microorganisms	Hospitalizations change	-0.0001	0.999	0.148	0.223	–	–	No significant correlation
Antibiotics post	Hospitalizations change	–	–	–	–	-0.389	0.699	No significant correlation
Antibiotics previous	Hospitalizations change	–	–	–	–	0.679	0.504	No significant correlation
UTI previous year	Hospitalizations change	0.332	0.005	-0.017	0.888	–	–	**Pearson significant,** Spearman not significant
Main bacteria specie	Hospitalizations change	-0.118	0.332	-0.040	0.741	–	–	No significant correlation
Etiology	Hospitalizations change	-0.028	0.816	0.053	0.660	–	–	No significant correlation
Age	Hospitalizations change	-0.202	0.093	-0.210	0.080	–	–	No significant correlation
Sex	Hospitalizations change	–	–	–	–	0.362	0.718	No significant correlation
Number of microorganisms	Emergencies admissions change	0.011	0.930	0.038	0.756	-0.389	–	No significant correlation
Antibiotics post	Emergencies admissions change	–	–	–	–	-1.955	0.056	No significant correlation
Antibiotics previous	Emergencies admissions change	–	–	–	–	0.100	0.921	No significant correlation
UTI previous year	Emergencies admissions change	0.584	1.34e-07	0.403	0.00059	–	–	**Pearson significant,** **Spearman significant**
Main bacteria specie	Emergencies admissions change	-0.071	0.562	-0.148	0.222	–	–	No significant correlation
Etiology	Emergencies admissions change	0.026	0.832	0.052	0.667	–	–	No significant correlation
Age	Emergencies admissions change	-0.210	0.081	-0.208	0.084	–	–	No significant correlation
Sex	Emergencies admissions change	–	–	–	–	-0.321	0.750	No significant correlation
UTI previous year	UTI Free	-0.007	0.958	0.019	0.878	–	–	No significant correlation

UTI, Urinary Tract Infection.

Bold words indicate statistical significance.

Regarding emergency admissions, most parameters again showed no significant associations. The only statistically significant association was observed between the number of UTIs in the previous year and emergency admissions. Both Pearson and Spearman correlation values were significant, with a Pearson correlation of 0.584 and a p-value of less than 0.0000001, and a Spearman correlation of 0.403 with a p-value of 0.00059.

No significant correlation was found between the number of UTIs in the previous year and achieving a UTI-free status after treatment.

## 4 Discussion

To our knowledge, this is the first study performed exclusively on the NB population to describe the effect of autovaccine-based immunoprophylaxis on UTI. As previously noted, UTIs are the most common infection in the NB population; 31% of patients with a new diagnosis of SCI were diagnosed with a UTI within the first year, and 21% required hospitalization (42). Due to the increased frequency and severity of infections, there is a higher risk of morbidity and mortality secondary to urosepsis and end-stage renal disease compared to the general population (4). Consequently, UTIs represent one of the most common morbidities and reason for the re-hospitalization of individuals with NB, posing a serious healthcare burden often resulting in significant morbidity and decreased quality of life (43). Moreover, people with NB often have other neurologic impairments such as limited mobility or cognitive dysfunction and face significant barriers to accessing health care—including transportation, geographic, and financial constraints (44). As a result, it is not unusual for people with NB and recurrent UTIs to self-manage and self-treat with antimicrobials based on urinary symptoms only (45).

Results from this observational, prospective, monocenter study evaluating the treatment outcomes of autovaccination in patients with NB who experience recurrent UTIs demonstrated a reduction in hospitalizations and emergency admissions. This decrease suggests a diminished burden of recurrent infections and a reduced need for medical interventions. Furthermore, the high overall satisfaction levels reported in patient questionnaires—particularly their willingness to continue treatment—lend support to these findings.

Previous studies have investigated the use of MV140 and autovaccine to prevent recurrent UTIs across various demographics, yielding encouraging outcomes in lowering infection rates and reducing reliance on antibiotics (11–33). However, data concerning patients with NB remains limited. A recent 2025 study included NB patients in its cohort, though they constituted a small percentage of the total participants and lacked specific disclosures or statistical analysis of this group (21). Our 2014 pilot study, which involved a cohort of 17 patients on MV140, did report explicit outcomes for this demographic (38). Overall, mirroring the current research in terms of age, gender, and inclusion criteria, the 2014 study also reported a reduction in hospitalizations (median of 1.5 to 0; p=0.036) and emergency admissions (median of 1 to 0; p=0.094), aligning with findings from the present study. Furthermore, the 2014 research demonstrated a significant decrease in leukocyte count (p<0.001), supporting the efficacy of autovaccination in enhancing the immune response against UTIs, particularly against pathogens such as *E. coli*, which was the most frequently identified organism in prior positive cultures, similar to our findings.

As mentioned, immunoprophylaxis had been tested previously in NB patients with recurrent UTIs (35–37), although the only two studies reporting efficacy data were conducted in NB patient subgroups that included paraplegic patients and those with chronic spinal cord injury (36, 37). Therefore, this is the first study to report efficacy data of immunoprophylaxis in patients with NB of diverse etiologies and recurrent UTIs. One possible reason for the lack of broader studies is that patients with NB represent a particularly complex demographic, often excluded from clinical trials due to the variability of their conditions and the challenges associated with standardizing treatment protocols. This exclusion is also evident in other studies on immunoprophylaxis for UTIs, where having NB often serves as a criterion for exclusion (11, 12). The rationale behind this exclusion stems from the distinct interpretation of UTIs within this group compared to the general population. Common symptoms, such as cloudy urine, are not deemed definitive for diagnosis (46), thus complicating infection assessments and necessitating more tailored approaches. A critical aspect of managing these patients includes avoiding prolonged antibiotic use, which can lead to resistance and adverse side effects (47). Immunoprophylaxis with autovaccines offers a valuable alternative, diminishing dependence on antibiotics and boosting the immune defense of the body against recurrent infections (6–9). Although this therapy may not consistently result in negative bacterial cultures in NB patients, it could markedly enhance the quality of life by reducing the frequency and severity of symptomatic infections, as evidenced by the significant reductions in hospitalizations and emergency admissions registered in the present study.

Furthermore, the generally positive response to the therapy, characterized by high levels of patient satisfaction and a willingness to undergo repeat treatments in the study cohort, supports these findings. However, it is important to consider those who were not satisfied with the immunoprophylaxis. Six out of 19 patients (32%) reported persistent cloudy or foul-smelling urine during catheterization and performed urine cultures, even though they were asymptomatic. Notably, three of these patients were concerned because the cultures were positive for a microorganism different from the one targeted by the autovaccine. Our interpretation of this scenario is that either these microorganisms were saprophytic in the urinary tract, or the vaccine induced a microbiological shift toward a less virulent strain incapable of causing symptoms. This situation highlights a gap in patient education, as some NB patients—particularly those practicing CIC—continue to self-treat asymptomatic bacteriuria.

While microbiological eradication is not the primary aim of immunoprophylaxis, it is noteworthy that some patients who developed UTIs post-treatment harbored different pathogens compared to those included in their original autovaccine. In the small number of symptomatic cases recorded at our institution, *Escherichia coli* and *Klebsiella pneumoniae* remained predominant. One possible explanation for this shift could be a vaccine-induced modulation of the urinary microbiome, which may suppress dominant uropathogens and permit the emergence of subdominant or less virulent strains. These observations underscore the need for more detailed microbiological follow-up studies to better understand pathogen dynamics post-immunoprophylaxis.

Regarding the statistical analysis of the association between autovaccination and changes in hospitalizations or emergency admissions, the findings indicate that the number of UTIs in the previous year is the most significant factor associated with variations in hospitalizations and emergency admissions. One plausible explanation is that patients who have experienced a greater number of hospitalizations and emergency admissions in the past tend to show a more noticeable difference following vaccination simply because they had a higher baseline of such events. In contrast, other clinical variables, such as the etiology of the infection, the specific bacterial species involved, antibiotic usage, and the age and sex of the patients, did not consistently predict outcomes post-autovaccination. Although studies involving larger population cohorts are needed, these findings support the notion that autovaccination can effectively reduce hospitalizations and emergency admissions across diverse patient populations, regardless of demographic factors, the bacterial species causing the infection, or prior antibiotic use. Further research is essential to refine predictive models for patient response to immunoprophylaxis and to solidify this understanding.

In managing patients with NB and recurrent UTIs, it is also crucial to consider the economic impact of the disease management. The World Health Organization (WHO) identifies vaccination as the most cost-effective strategy for controlling infectious diseases, a principle that holds promise for reducing the economic burden associated with recurrent UTIs (48). Although the current study did not incorporate specific economic data, it is notable that the observed reduction in hospitalizations and emergency admissions due to vaccination likely exceeds the costs of the treatment. Further evidence of the economic benefits of vaccination comes from three studies focused on the prevention of recurrent UTIs (23, 25, 49). Carrión-López et al. demonstrated statistically significant cost savings (p < 0.02) after MV140 treatment in women with recurrent UTIs, with a reduction in healthcare expenditure per patient per year from a mean of €1,001.1 to €497.1 (23). Two other studies also focused on recurrent UTIs—one in women and the other in a mixed-gender population—found that treatment with MV140 autovaccination was not only more effective (25) but also associated with lower healthcare costs compared to standard antibiotic treatment (25, 49). Given these findings, while the current data underscores the potential of vaccination to diminish healthcare utilization and associated costs significantly, further studies are needed to comprehensively assess the economic impact and optimize care for patients with neurogenic bladders and recurrent UTIs.

One of the aspects to highlight in the present investigation is the lack of previous scientific evidence; therefore, we had to establish a treatment protocol. Although we designed the study following the approach used in other studies performed in general populations, where the treatment scheme consisted of a 3-month treatment period followed by 9 months without treatment (11–33). However, once our study ended, based on the UTI-free rate obtained in this analysis (from 69.12% at 3 months to 20.59% at 12 months), and considering the increased risk factors in the NB population, we thought to change our daily clinical practice to administer the autovaccine once every 6 months for at least 3 years. Further research will be necessary to refine the optimal dosage regimen and recall strategies to maintain long-term protection against recurrent infections.

To interpret the findings of this study, we must acknowledge its limitations. The absence of a control group limits direct comparisons to standard preventive measures, which limits the ability to draw definitive causal conclusions. However, in our opinion, given the exploratory nature of the study and ethical considerations in delaying potentially beneficial treatment in a high-risk population, a randomized design was not feasible. Instead, we adopted a within-subject comparison framework. Nevertheless, future controlled, multicenter trials are essential to confirm these findings. Moreover, the exclusion of immunocompromised patients, including those with diabetes, was initially implemented to reduce variability in immune response and avoid confounding results. However, emerging evidence supports the efficacy of MV140 even in immunosuppressed populations, such as those with autoimmune conditions and renal transplantation. Future studies should aim to include this subgroup to enhance generalizability and assess outcomes in real-world NB cohorts. Additionally, as a monocenter study, the findings may not be fully generalizable to broader patient populations. Future multicenter studies with larger cohorts and standardized assessment tools will be needed to confirm these results and provide more robust evidence on the efficacy of autovaccination in NB patients.

In conclusion, the results of the present study support the use of autovaccine as an effective measure to prevent hospitalizations and emergency department admissions in patients with NB presenting with UTIs. The majority of patients perceive its efficacy as positive, reinforcing its potential role in clinical practice. Despite these promising results, the complexity of NB conditions and the variability in patient responses call for further investigative efforts. Larger, more diverse studies are required to validate these findings across broader demographics, define the need for revaccination, and refine economic evaluations, ensuring that autovaccination can be effectively integrated into standard care protocols. By continuing to explore these avenues, healthcare providers can better address the nuanced needs of this patient population, potentially transforming the landscape of treatment for recurrent UTIs and minimizing the economic impact on health systems.

## Data Availability

The raw data supporting the conclusions of this article will be made available by the authors, without undue reservation.
